# Simultaneous Vector Bend and Temperature Sensing Based on a Polymer and Silica Optical Fibre Grating Pair

**DOI:** 10.3390/s18103507

**Published:** 2018-10-17

**Authors:** Binbin Yan, Guoqiang Liu, Jun He, Yanhua Luo, Liwei Yang, Haifeng Qi, Xinzhu Sang, Kuiru Wang, Chongxiu Yu, Jinhui Yuan, Gang-Ding Peng

**Affiliations:** 1 State Key Laboratory of Information Photonics and Optical Communications, Beijing University of Posts and Telecommunications, Beijing 100876, China; yanbinbin@bupt.edu.cn (B.Y.); guoqiangbupt@foxmail.com (G.L.); xzsang@bupt.edu.cn (X.S.); krwang@bupt.edu.cn (K.W.); cxyu@bupt.edu.cn (C.Y.); yuanjinhui81@163.com (J.Y.); 2 Key Laboratory of Optoelectronic Devices and Systems of Ministry of Education and Guangdong Province, Shenzhen University, Shenzhen 518060, China; hejun07@szu.edu.cn; 3 Photonics & Optical Communications, School of Electrical Engineering, University of New South Wales, Sydney, NSW 2052, Australia; g.peng@unsw.edu.au; 4 College of Information and Electrical Engineering, China Agricultural University, Beijing 100083, China; yangliwei@cau.edu.cn; 5 Laser Institute, QiLu University of Technology (Shandong Academy of Sciences), Jinan 250014, China; qihf@sdlaser.cn

**Keywords:** polymer fibre grating, silica fibre grating, fibre Bragg grating, vector bend sensing, temperature sensing, simultaneous sensing

## Abstract

The bending response of polymer optical fibre Bragg grating (POFBG) and silica optical fibre Bragg grating (SOFBG) mounted on a brass beam have been systematically studied and compared. The results indicate that POFBG has higher (almost twice as much) bend sensitivity than SOFBG. Based on the difference between the bend and temperature sensitivity of POFBG and SOFBG, a new method of measuring vector bend and temperature simultaneously was proposed by using a hybrid sensor head with series connection of one POFBG and one SOFBG with different Bragg wavelengths. It provides high sensitivity and resolution for sensing bend and temperature changes simultaneously and independently. The proposed sensor can find some applications in the fields where high sensitivity for both bend and temperature measurements are required.

## 1. Introduction

Sensing has become a key enabling technology in many areas, from entertainment to health and transportation. Among many advanced sensors, miniaturization, high sensitivity and remote sensing are the most important requirements, which optic fibre sensing can effectively meet. Among the various fiber sensors fibre grating-based sensing has proven to be more promising in many photonic applications [1,2]. More recently, the fibre gratings based on polymer optical fibre (POF) have attracted more and more interest due to the rather different material properties of polymers compared with silica, which provide advantages in certain applications [2,3]. Since the first POF gratings were inscribed in 1999, there have been numerous studies of their applications for sensing temperature [4], strain [5], stress [6], humidity [7], force [8], pressure [9], bend [10], refractive index [11], erythrocyte concentration [12], etc. [2,3]. One very popular topic in the POF grating-based sensing field nowadays is the discriminant measurement of multiple parameters, which has been achieved based on POF gratings with different schemes to some degree. For example, polymer and silica fibre Bragg dual gratings [13], few-mode polymer optical fibre gratings [13], and polymer fibre Bragg grating (FBG) pairs with one etched and on unetched polymer grating [14] have been used for the simultaneous sensing of temperature and strain. Recently, simultaneous humidity and temperature sensing have also been achieved with a Zeonex/poly (methyl methacrylate) (PMMA) micro-structured polymer optical fiber (mPOF) Bragg grating pair [15]. However, there is no report of the simultaneous measurement of bend and temperature based on POF gratings, although there have been many reports based on silica-based fibre gratings, like the titled FBG [16,17], long-period fibre gratings [18,19,20], the eccentric core FBG cascaded with a Fabry-Perot cavity [21], few-mode PCF formed Mach–Zehnder interferometer and an embedded FBG [22], long period grating and fiber Bragg grating pairs [23].

So far, there are only a few reports on bend sensing based on POF gratings. An eccentric-cored POFBG has a vector bend sensitivity of 63.3 pm/m^−1^ [10,24], and a sensitivity of −28.2 pm/m^−1^ has been observed in D-shaped POF gratings [25]. In our recent work, a vector bend sensor with a high sensitivity of 937 pm/m^−1^ has been obtained based on a polymer and silica fibre Bragg grating pair for the greater enhancement of the sensitivity than a single fibre grating [26]. However, in a lot of the practical circumstances, the bend state and temperature always change simultaneously and independently. Here, we propose to use the polymer and silica fibre Bragg grating pair for the simultaneous vector bend and temperature sensing. The mounting influence and stability have also been further studied and analysed.

## 2. Principle

### 2.1. Bend Sensing

**Figure 1:**
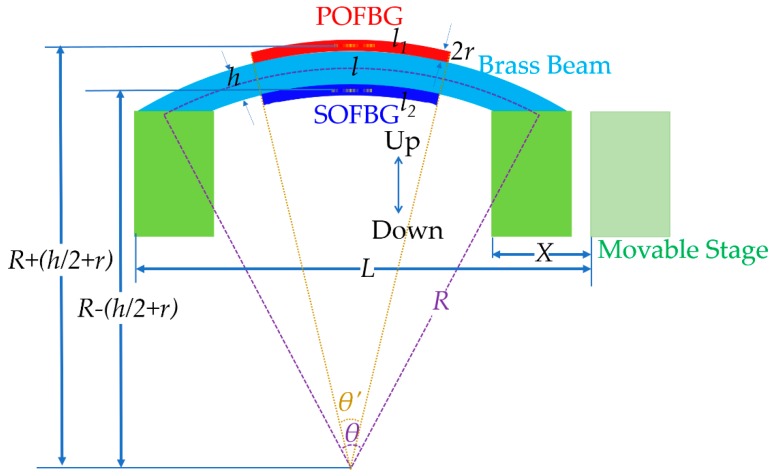
A typical experimental scheme for the vector bend sensing of brass beam with POFBG and SOFBG.

When a fibre grating is bent, its grating period and effective refractive index will vary. If the grating is fixed on a brass beam, the fibre grating will be deformed, as shown in Figure 1, when the beam is bent. Seen from Figure 1, the response of the fibre grating is expressed in the form of stress. It is obvious that if the fibre grating is located above or below the neutral axis of beam, the fibre grating will be in extension or compression during bending. For example, if the fibre grating is bent upwards, the fibre gratings fixed on the upper surface of the beam will be extended, while that on the lower surface will be compressed.

As shown in Figure 1, when the right end of the beam is moved towards the left side, the curvature of beam (*C*) can be approximately expressed by [27]:

(1) $$\pm C=\frac{1}{R}≅\sqrt{24X/L^{3}},$$

where *R* is the radius of the bent beam, *X* is the movement distance of the movable stage, and *L* is the length of beam, the ‘±’ sign of the curvature indicates the bend direction (upwards bending is positive while downwards is negative, when the fibre grating is mounted above the neutral axis.). The center angle of the bending (*θ*) for the beam is given by:

(2) $$\theta =\frac{L}{R}.$$

Similarly, the centre angle of the bending (*θ*′) for the fibre is given by:

(3) $$\theta^{\prime }=\frac{l}{R}=\frac{l_{1}}{R+\left(\frac{h}{2}+r\right)}=\frac{l_{2}}{R-\left(\frac{h}{2}+r\right)}.$$

where *h* is the thickness of the beam and *r* is the radius of the fibre, *l* is the length of fibre before bending, and *l*_1_ and *l*_2_ are the length of the fibre on the upper surface and lower surface of the beam after bending, respectively. Therefore, when the beam is bent upwards, the strain of the fibre gratings on the upper surface will be given by:

(4) $$\varepsilon_{up,up}=\frac{l_{1}-l}{l}=\frac{\left\{\theta^{\prime }\left[R+\left(\frac{h}{2}+r\right)\right]-l\right\}}{l}=\frac{\theta^{\prime }}{l}\left(\frac{h}{2}+r\right)=\frac{1}{R}\left(\frac{h}{2}+r\right),$$

and the strain of the fibre gratings on the lower surface will be given by:

(5) $$\varepsilon_{up,low}=\frac{l_{2}-l}{l}=\frac{\left\{\theta^{\prime }\left[R-\left(\frac{h}{2}+r\right)\right]-l\right\}}{l}=-\frac{\theta^{\prime }}{l}\left(\frac{h}{2}+r\right)=-\frac{1}{R}\left(\frac{h}{2}+r\right).$$

Similarly, when the beam is bent downwards, the strain of the fibre gratings on the upper surface will be given by:

(6) $$\varepsilon_{down,up}=\frac{\left\{\theta^{\prime }\left[R-\left(\frac{h}{2}+r\right)\right]-l\right\}}{l}=-\frac{\theta^{\prime }}{l}\left(\frac{h}{2}+r\right)=-\frac{1}{R}\left(\frac{h}{2}+r\right);$$

and the strain of the fibre gratings on the lower surface will be given by:

(7) $$\varepsilon_{down,low}=\frac{\left\{\theta^{\prime }\left[R+\left(\frac{h}{2}+r\right)\right]-l\right\}}{l}=\frac{\theta^{\prime }}{l}\left(\frac{h}{2}+r\right)=\frac{1}{R}\left(\frac{h}{2}+r\right).$$

Combining Equations (1)–(7), there will be:

(8) $$\varepsilon_{up,up}=\varepsilon_{down,low}=\frac{1}{R}\left(\frac{h}{2}+r\right)=C\left(\frac{h}{2}+r\right)$$

and:

(9) $$\varepsilon_{up,low}=\varepsilon_{down,up}=-\frac{1}{R}\left(\frac{h}{2}+r\right)=-C\left(\frac{h}{2}+r\right).$$

If the strain is homogeneous and isotropic, the shift of the Bragg wavelength of fibre grating due to the strain change can simply be given by [28]:

(10) $$\Delta \lambda_{B}=\lambda_{B}\left(1-p_{e}\right)\cdot \varepsilon ,$$

where *p_e_* is an effective photoelastic constant defined by:

(11) $$p_{e}=\frac{n_{eff}^{2}}{2}\left[p_{12}-v\left(p_{11}+p_{12}\right)\right].$$

where *p_ij_* are the photoelastic constants of the strain optic tensor and *v* is the Poisson ratio.

According to Equations (8)–(10), the shift of the Bragg wavelength of fibre grating will be given by:

(12) $$\Delta \lambda_{B}=\lambda_{B}\left(1-p_{e}\right)\left(\frac{h}{2}+r\right)C=K_{C}C$$

where $K_{C}=\lambda_{B}\left(1-p_{e}\right)\left(\frac{h}{2}+r\right)$ can be taken as the bend sensitivity, which indicates that the sensitivity is not only related with photoelastic constants of the fibre but also the dimension of the fibre used (The influence of the fibre dimension can often be neglected if the beam dimension (*h*/*2*) is often far larger than the fibre dimension (*r*)). Please note that the ‘*C*’ will have the ‘±’ sign, which indicates the relative direction between the mount position of fibre grating and bend direction. If they are the same, it will be positive. On the contrary, it will be negative.

### 2.2. Temperature Sensing

The *Bragg* wavelength shift Δ*λ_B_* of fibre gratings with temperature change of Δ*T* can be given by [29]:

(13) $$\Delta \lambda_{B}=\lambda_{B}\left(\alpha_{f}+\xi \right)\;\Delta T$$

where $\alpha_{f}$ is the thermal expansion coefficient and ξ is the thermo-optic coefficient of the SOFBG and POFBG as listed in Table 1.

As the fibre grating is bonded onto a beam, the thermal expansion of the beam causes a change in the grating period. As the thermal expansion coefficient of brass is much larger than that of SOFBG, considering only the effect of longitudinal strain applied to the SOFBG due to the beam, the temperature induced wavelength shift Δ*λ_B_* of SOFBG can further be written as [30]:

(14) $$\Delta \lambda_{B}=\lambda_{B}\left[\left(1-p_{e}\right)\alpha_{b}+\xi \right]\Delta T$$

where $\alpha_{b}$ is the thermal expansion coefficient of the brass. However, it is not applicable for POFBG, as the thermal expansion coefficient of beam is much less than that of POFBG as listed in Table 1. So the thermal expansion of the beam has negligible effect upon the temperature response of POFBG, and Equation (13) will still be satisfied for POFBG, where similar property has been verified by the report before [31].

## 3. Experiment

### 3.1. POFBG & SOFBG Sample

**Figure 2:**
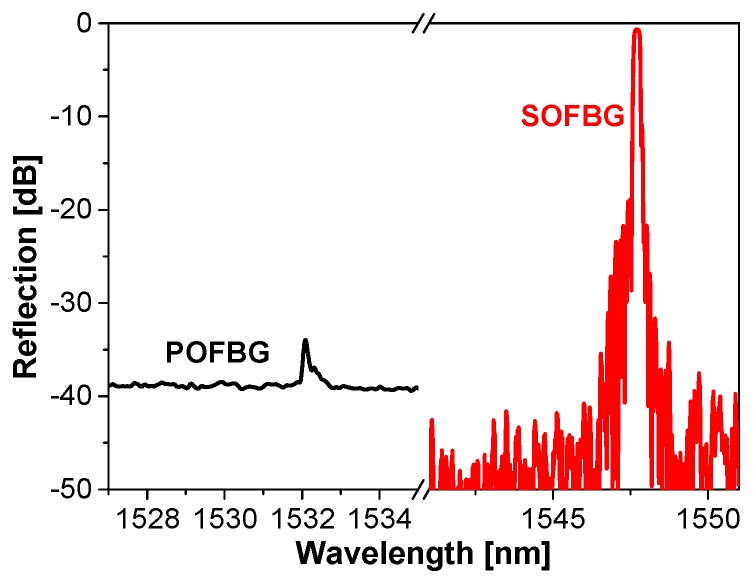
Reflection spectra of typical POFBG and SOFBG at 25 °C.

POFBG and SOFBG used for sensing experiments are fabricated with phase mask techniques. POFBG is inscribed using a 50 mW 325 nm Kimmon He–Cd laser and the grating region is about 10 mm long, while SOFBG was made using a 248 nm KrF excimer laser (200 Hz, 14 mJ) and the grating region is also about 10 mm long. Their reflection spectra at room temperature are shown in Figure 2, which are measured with an optical vector analyzer (OVA e-4000NF, LUNA, Roanoke, VA, USA) integrated a tunable laser (working wavelength: 1525–1605 nm) and a detector. The wavelength resolution is 2.6 pm. POFBG is fabricated in the single mode PMMA POF made by UNSW (diameter of POF: *d_POF_* ≈ 250 μm), with its Bragg wavelength at 1532.087 nm, while SOFBG in the standard single mode fibre (SMF) (diameter of SOF: *d_SOF_* = 125 μm), with its Bragg wavelength at 1547.715 nm.

### 3.2. Sensing Experimental Setup

POFBG with length of approximately 10 cm is used and one end of POF is pigtailed with a silica SMF using an UV curable glue [35], while SOFBG is directly fused with SMF. Both end parts of the fibre gratings are fixed on the upper surface of the brass beam with epoxy glue. The thickness and length of the beam is ~0.5 mm and 16 cm, and the whole fibre is stuck tightly to the beam with sticky tape unless otherwise stated. Then one end of the beam is mounted on the fixed metal block and the other end on the movable stages. It is intended that both the fibre gratings and the beam be mounted with zero strain, but they required to be kept straight. Bend is generated by squeezing of the beam through the movement of the movable stage. The reflection spectrum of fibre gratings during the bending process is also monitored and recorded by the optical vector analyzer.

The temperature sensing of POFBG (in the free state) was performed with similar experimental setup reported before [36], where the sensing part of the POFBG was put into an oven, while the SOFBG (in the free state) was put into a water bath. For SOFBG on the beam, two ends of SOFBG are fixed with epoxy glue (minor strain is applied) and it is immersed into the water bath together with the beam. Temperature was controlled by a proportional–integral–derivative controller with temperature accuracy of ±1 °C. The real temperature was read from a mercury thermometer with ±0.02 °C accuracy. All the sensing points are recorded with 5 min stabilization after the targeted temperature setting was reached.

## 4. Results and Discussion

### 4.1. Vector Bend Response of POFBG & SOFBG

**Figure 3:**
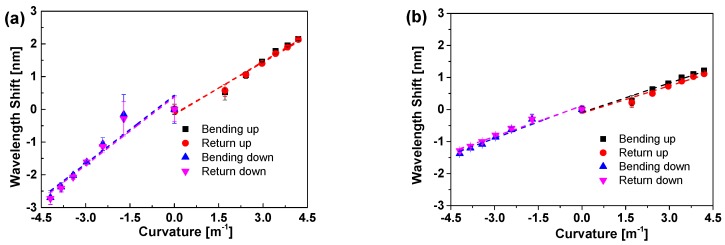
Bend response of POFBG (**a**) and SOFBG (**b**) on the upper surface of brass beam vs. the curvature, when the beam is bent upwards or downwards.

The bend response of POFBG and SOFBG for the upwards and downwards bending have been tested individually. The Bragg wavelength shift of POFBG and SOFBG responding to the bend are plotted in Figure 3a,b, respectively, when they are in bending and unbending. As seen from Figure 3a,b, the wavelength shift response to the upwards and downwards bending are different for both POFBG and SOFBG due to the different pre-strain applied. Especially, the difference for POFBG is larger than that for SOFBG, which might be due to the viscoelasticity of the POF.

According to Equation (12), the Bragg wavelength almost linearly shifts responding to the bend. Therefore, the experimental data are linearly fitted as the dash lines shown in Figure 3 and the details of the linear fitting results are listed in Table 2. The low residual sum of squares and high adjustment R^2^ indicates well linear fitting, which is consistent with Equation (12). The sensitivity of POFBG is larger than that of SOFBG, almost two times, although the residual sum squares for POFBG is a bit larger than that of SOFBG. According to Equation (12), the larger sensitivity of POFBG may be attributed to two reasons: (1) the fibre diameter of POFBG is almost twice of that of SOFBG; (2) The effective photoelastic constant of POFBG is much less than that of SOFBG [37,38]. Taking into account the linear fit to experimentally measured data, the sensitivities are 0.533 nm/m^−1^ and 0.695 nm/m^−1^ for POFBG in the upwards and downwards bending, while those are 0.295 nm/m^−1^ and 0.329 nm/m^−1^ for SOFBG, respectively. There are some differences in intercepts of the upwards and downwards bending attributed to the different pre-strain applied as well as the viscoelasticity of the POF [39].

#### 4.1.1. Mounting Influence

**Figure 4:**
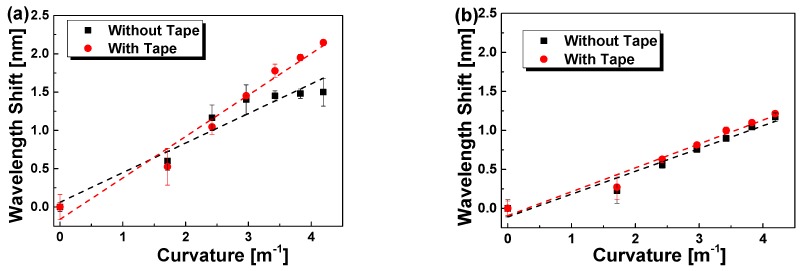
Bend response of POFBG (**a**) and SOFBG (**b**) on the upper surface of brass beam without and with sticky tape fixing vs. the curvature, when the beam is bent upwards.

We have also studied the mounting influence of the sticky tape over the middle part of the fibre gratings, besides the fixing of both ends of the fibre gratings on the upper surface of the beam. The Bragg wavelength shifts of POFBG and SOFBG responding to the bend are plotted in Figure 4a,b without and with sticky tape fixing, respectively. The experimental data are linearly fitted, and the fitting results are listed in Table 3. Seen from Figure 4a, without the sticky tape fixing, the response of the POFBG is greatly slower than that with sticky tape, which is more evidently at the large curvature region. The fixing with sticky tape has great influence upon POFBG compared with SOFBG in Figure 4b, although similar phenomenon is observed for SOFBG. 

The main reason may be the non-uniformity straining in the bending process if there is no sticky tape fixing. Compared with commercial SMF, the homemade POFBG has more intrinsic non-uniformity. In addition, the viscoelasticity of the POF may be another reason. Especially, with sticky tape fixing, the residual sum of the squares is less. Therefore, it is necessary to fix the whole fibre gratings onto the beam for the higher and accurate sensing.

#### 4.1.2. Viscoelasticity Influence

**Figure 5:**
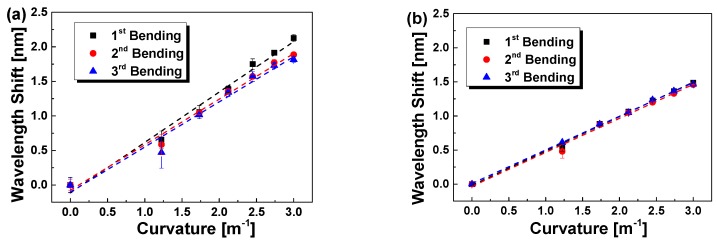
Bend response of POFBG (**a**) and SOFBG (**b**) on the upper surface of brass vs. the curvature in repeated upwards bending, where the beam length changed to 20 cm.

The Bragg wavelength shift of POFBG and SOFBG response in several bending upwards are plotted in Figure 5a,b, respectively. The experimental data are linearly fitted, and the fitting results are listed in Table 4. Compared with the short beam (16 cm) in Figure 3 and Figure 4, the bend sensitivity with the long beam (20 cm) is evidently increased as shown in Table 4, although from Equation (12) the sensitivity almost has no relationship with the length of the beam. It is influenced by the neglected 3rd terms in Taylor expansion for the calculation of the curvature from the displacement [27], where once the beam length increased, the true *C* will be increased, resulting in more shift of the Bragg wavelength. Seen from Figure 5 and Table 4, the intercepts for POFBG have a larger difference than those for SOFBG. The POFBG sensitivity varies from 0.654 to 0.729 nm/m^−1^ while that of SOFBG is much less, and varies from 0.493 to 0.504 nm/m^−1^. Especially, the residual sum of squares for SOFBG is less than 0.015 nm and that for POFBG is over than 0.032 nm. Such results indicate that the repeatability for SOFBG is higher than that of POFBG, in single/multi repeated bending processes, which may be due to the viscoelasticity of the POFBG.

### 4.2. Temperature Response of POFBG & SOFBG

**Figure 6:**
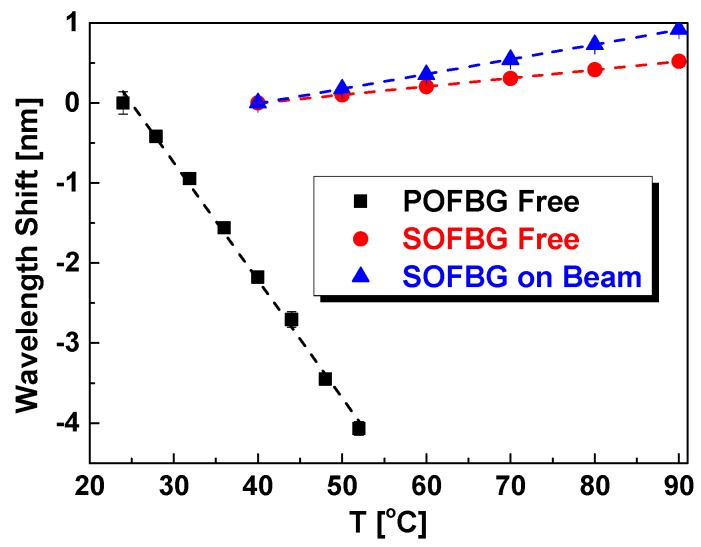
Bragg wavelength shift of POFBG and SOFBG vs. temperature.

The thermal responses of POFBG and SOFBG are measured and the results are plotted in Figure 6. The dashed line is the linear fitting of experimental data and the fitting parameters are listed in Table 5. Seen from Figure 6, the Bragg wavelength shift of POFBG decreases linearly with temperature due to the large negative thermo-optic coefficient [40], while that of SOFBG increases linearly. Seen from Table 5, the temperature sensitivity of POFBG (−0.147 nm/°C) is much higher than that of SOFBG (0.010 nm/°C). If the POFBG is bonded to the brass beam, the temperature sensitivity will almost keep the same due to the larger thermal expansion coefficient of POF over brass. However, that of SOFBG will have great influence by the beam as shown in Figure 6, where the sensitivity of the SOFBG was increased to 0.018 nm/°C, due to the larger thermal expansion coefficient of brass than that of SOFBG.

### 4.3. Simultaneous Bend and Temperature Sensing

Since the absolute Bragg wavelength of the fibre grating sensors is dependent on both bend and temperature effects as shown above, a single measurement of Bragg wavelength shift cannot distinguish between the effects of bend and temperature, but in most circumstances, they can change simultaneously and independently. Thus the discriminating technique involving FBG sensors is of great importance. As demonstrated previously, the bend and temperature sensitivity of POFBG is quite different from that of SOFBG. Furthermore, the temperature coefficient for POFBG is negative, which is a totally different response compared with that of SOFBG. Hence, we propose a hybrid sensor with series connection of one POFBG and one SOFBG with different Bragg wavelengths. 

The shift of Bragg wavelength Δ*λ_B_* due to the combined response to the change of both bend and temperature, Δ*C* and Δ*T*, is given by:

(15) $$\left[\begin{array}{c} \Delta \lambda_{P}\\ \Delta \lambda_{S} \end{array}\right]=\left[\begin{array}{cc} K_{PC} & K_{PT}\\ K_{SC} & K_{ST} \end{array}\right]\left[\begin{array}{c} \Delta C\\ \Delta T \end{array}\right]$$

where *K_PC_* and *K_SC_* are the bend sensitivities of POFBG and SOFBG, while *K_PT_* and *K_ST_* are the temperature sensitivities, respectively. By inversing the matrix in Equation (15), the information of Δ*C* and Δ*T* can be recovered,

(16) $$\left[\begin{array}{c} \Delta C\\ \Delta T \end{array}\right]=\frac{1}{K_{PC}K_{ST}-K_{SC}K_{PT}}\left[\begin{array}{cc} K_{ST} & -K_{PT}\\ -K_{SC} & K_{PC} \end{array}\right]\left[\begin{array}{c} \Delta \lambda_{P}\\ \Delta \lambda_{S} \end{array}\right]$$

From our previous results, the sensitivities for POFBG and SOFBG in difference cases are summarized and listed in Table 6. As listed in Table 6, due to the difference of the sensitivity for the upwards and downwards bending as well as the mounting, there are eight cases of the sensing scheme, but the sensing equation can be simplified into four sets. Take the scheme of the upper mounting of POFBG and SOFBG in upwards bending as an example. Assuming that the operating wavelength for POFBG and SOFBG are *λ_POFBG_* = 1530 nm and *λ_SOFBG_* = 1550 nm, the bend and temperature change, Δ*C* and Δ*T*, can be given when the Bragg wavelength shift is known as:

(17) $$\left[\begin{array}{c} \Delta C\\ \Delta T \end{array}\right]=\left[\begin{array}{cc} 0.340 & 2.776\\ -5.570 & 10.064 \end{array}\right]\left[\begin{array}{c} \Delta \lambda_{P}\\ \Delta \lambda_{S} \end{array}\right]$$

As a result, simultaneous measurement of bend and temperature by using hybrid POFBG and SOFBG sensor head can be easily accomplished. Given the wavelength resolution of the optical vector analyzer of 2.6 pm, according to Equation (17), the resolution in terms of temperature and bend sensing is up to 0.012 °C and 0.008 m^−1^, respectively.

## 5. Conclusions

Vector bend sensing based POFBG has been studied compared with SOFBG. The result indicates that the POFBG has higher sensitivity than SOFBG. Especially, based on the difference of the bend and temperature sensitivity between POFBG and SOFBG, a simple and effective scheme for simultaneous measurement of bend and temperature by using a hybrid POFBG and SOFBG sensor head has been proposed for future applications in fibre sensing fields. Given such a simultaneous measurement of these parameters, the proposed sensor could find applications in areas like human joint monitoring in the bio-medical field, robot monitoring in the artificial intelligence field, etc. where high sensitivity bend and temperature measurements are both required.

## Figures and Tables

**Table 1:** Relevant parameters of SOFBG and POFBG [32,33,34].

	Parameter	Thermal Expansion Coefficient (ξ)	Thermal Expansion Coefficient (*α*)
Sample		/°C	/°C
**SOFBG**		~8.6 × 10^−6^	0.55 × 10^−6^
**POFBG**		−100 × 10^−6^	50 × 10^−6^
**Brass**		-	19 × 10^−6^

**Table 2:** The linear fitting results of POFBG and SOFBG response to the bend.

FBG	Fitting	Direction	Slope	Intercept	Residual Sum of Squares	Adj. *R*^2^	*C*	*K_C_*
			nm/m^−1^	nm	nm		m^−1^	nm/m^−1^
**POFBG**	Bending	Up	0.541	−0.162	0.105	0.966	+	0.533
	Return	Up	0.525	−0.136	0.067	0.977		
	Bending	Down	0.696	0.427	0.654	0.882	−	0.695
	Return	Down	0.695	0.377	0.695	0.907		
**SOFBG**	Bending	Up	0.306	−0.093	0.037	0.963	+	0.295
	Return	Up	0.284	−0.115	0.048	0.977		
	Bending	Down	0.340	0.122	0.054	0.957	−	0.329
	Return	Down	0.318	0.111	0.042	0.961		

**Table 3:** The linear fitting results of POFBG and SOFBG response to the bend with and without sticky tape fixing.

FBG	Sticky Tape	Slope (*K_C_*)	Intercept	Residual Sum of Squares	Adj. *R*^2^
		nm/m^−1^	nm	nm	
**POFBG**	×	0.386	0.062	0.125	0.924
	√	0.541	−0.162	0.105	0.966
**SOFBG**	×	0.292	−0.110	0.045	0.952
	√	0.306	−0.093	0.037	0.963

**Table 4:** The linear fitting results of POFBG and SOFBG response to the repeated upwards bending.

FBG	Cycle	Slope (*K_C_*, nm/m^−1^)	Intercept (nm)	Residual Sum of Squares (nm)	Adj. *R*^2^
**POFBG**	1st	0.729	−0.113	0.050	0.983
	2nd	0.660	−0.076	0.032	0.986
	3rd	0.654	−0.104	0.075	0.968
**SOFBG**	1st	0.504	−0.018	0.005	0.996
	2nd	0.499	−0.032	0.013	0.990
	3rd	0.493	0.009	0.001	0.999

**Table 5:** The linear fitting results of thermal response of POFBG and SOFBG.

Grating Type	Slope (nm/°C)	Intercept (nm)	Residual Sum of Squares (nm)	Adj. *R*^2^
**POFBG free**	−0.147	3.668	0.052	0.996
**SOFBG free**	0.010	−0.420	0.000	1.000
**SOFBG on beam**	0.018	−0.746	0.000	1.000

**Table 6:** Parameters of the simultaneous vector bending and temperature sensing for different schemes.

Mounting Case		Bending Direction	*K_PT_*	*K_ST_*	*K_PC,up_*	*K_PC,down_*	*K_SC,up_*	*K_SC,down_*
POFBG	SOFBG		−0.147	0.018	0.533	0.695	0.295	0.329
			nm/°C	nm/°C	nm/m^−1^	nm/m^−1^	nm/m^−1^	nm/m^−1^
Upper	Upper	Upwards	√	√	√		√	
Upper	Upper	Downwards	√	√		√		√
Lower	Lower	Upwards	√	√		√		√
Lower	Lower	Downwards	√	√	√		√	
Upper	Lower	Upwards	√	√	√			√
Upper	Lower	Downwards	√	√		√	√	
Lower	Upper	Upwards	√	√		√	√	
Lower	Upper	Downwards	√	√	√			√
